# Supplementary material for: Systematic and meta-based evaluation of the relationship between the built environment and physical activity behaviors among older adults
Source: PeerJ. 2023 Sep 25;11:e16173. doi: 10.7717/peerj.16173 (PMC10538293; doi:10.7717/peerj.16173)
Supplement: Supplemental Information 1 [file peerj-11-16173-s001.docx]

**Appendix A.** Schematic diagram of ecological model of health promotion.
