# Supplementary material for: Systematic and meta-based evaluation of the relationship between the built environment and physical activity behaviors among older adults
Source: PeerJ. 2023 Sep 25;11:e16173. doi: 10.7717/peerj.16173 (PMC10538293; doi:10.7717/peerj.16173)
Supplement: Supplemental Information 2 [file peerj-11-16173-s002.docx]

| **Appendix B. Examples of built environment questionnaire** | | | |
| --- | --- | --- | --- |
| **Questionaire Name** | **Nation** | **Year** | **Items** |
| PWES | Australia | 2004 | Convenience, aesthetics, service accessibility, traffic problems |
| Irvine-Minnesota Inventory | The U.S.A. | 2006 | Including 176 subjective evaluation items |
| IPAQ-E | Sweden | 2003 | Density, accessibility, aesthetics, social environment, street connectivity, neighborhood safety, etc |
| NEWS-A | The U.S.A. | 2006 | Density, mixed land use density, street connectivity, aesthetics, traffic safety, etc |
