# Supplementary material for: Systematic and meta-based evaluation of the relationship between the built environment and physical activity behaviors among older adults
Source: PeerJ. 2023 Sep 25;11:e16173. doi: 10.7717/peerj.16173 (PMC10538293; doi:10.7717/peerj.16173)
Supplement: Supplemental Information 3 [file peerj-11-16173-s003.docx]

| **Appendix C.** PubMed search strategy | |
| --- | --- |
| #1 | Search (Exercise[MeSH Terms]) OR Exercise[Title/Abstract] (435,091) |
| #2 | Search Physical activity[Title/Abstract] (137,977) |
| #3 | Search Fitness[Title/Abstract] (91,284) |
| #4 | Search Physical exercise[Title/Abstract] (19,238) |
| #5 | Search (Sports[MeSH Terms]) OR Sports[Title/Abstract] (249,064) |
| #6 | #1 OR #2 OR #3 OR #4 OR #5 (673,169) |
| #7 | Search (Aged[MeSH Terms]) OR Aged[Title/Abstract] (3,869,067) |
| #8 | Search Elderly[Title/Abstract] (282,389) |
| #9 | Search Older adults[Title/Abstract] (104,687) |
| #10 | Search Seniors[Title/Abstract] (8,583) |
| #11 | Search (Geriatrics[MeSH Terms]) OR Geriatrics[Title/Abstract] (37,828) |
| #12 | Search Aging[Title/Abstract] (233,510) |
| #13 | Search Age-related[Title/Abstract] (113,336) |
| #14 | #7 OR #8 OR #9 OR #10 OR #11 OR #12 OR #13 (4,129,568) |
| #15 | Search Environment[Title/Abstract] OR Environmental factors[Title/Abstract] OR Aesthetics[Title/Abstract] OR Virescence[Title/Abstract] OR Pedestrian facilities[Title/Abstract] OR Commercial facilities[Title/Abstract] OR Density[Title/Abstract] OR Accessibility[Title/Abstract] OR Traffic safety[Title/Abstract] OR Mixed land use[Title/Abstract] OR Crime rate[Title/Abstract] (1,452,459) |
| #16 | Search Effect[Title/Abstract] OR Influence[Title/Abstract] OR Correlate*[Title/Abstract] OR Risk factor[Title/Abstract] OR Protective factor[Title/Abstract] (5,795,182) |
| #17 | Search ("2010/01/01"[Date - Publication] : "2021/01/01"[Date - Publication]) (12,294,434) |
| #18 | #6 AND #14 AND #15 AND #16 AND #17 (2,947) |
| #19 | Search #18 NOT (("systematic review"[Filter]) AND ("review"[Filter]) AND ("meta-analysis"[Filter])) (207) |
