# Supplementary material for: Systematic and meta-based evaluation of the relationship between the built environment and physical activity behaviors among older adults
Source: PeerJ. 2023 Sep 25;11:e16173. doi: 10.7717/peerj.16173 (PMC10538293; doi:10.7717/peerj.16173)
Supplement: Supplemental Information 5 [file peerj-11-16173-s005.docx]

**Systematic Review and Meta-Analysis Rationale**

1. The rationale for conducting the systematic review / meta-analysis;

A rapid development of social economy and science and technology, such as human transportation and travel, has contributed to making production and living more convenient, but at the same time, it has objectively contributed to people's lack of physical activity (LPA), a factor that has caused more and more people to get into a sub-health or even a disease state. The relationship between the built environment and the physical activity behaviors (PABs) of the elderly has received widespread attention from the academic community, but there have not been more consistent research results. Moreover, to the best of our knowledge, the relationship between the built environment and PABs has been well explored in children and teenagers, while the evidence between such exposures in older adults was limited. In view of the above, this paper conducted a systematic evaluation in which a meta-analysis was used to quantitatively synthesize multiple original research results.

1. The contribution that it makes to knowledge in light of previously published related reports, including other meta-analyses and systematic reviews

It should be pointed out that this was the first study that used a comprehensive search for cross-sectional studies on the effect of the built environment on PABs of the elderly and the The Social-ecological Model (SEM) framework was innovatively introduced in to evaluate the influence of the built environment on the PABs of the elder population. Simultaneously, meta-based analysis based on PRISMA guidelines was applied to this study, which had a wide horizon of current hotspots and can quantitatively reflect the research status in the field and we anticipated this study would inspire future research in this field.
